# Boosting the immunogenicity of the CoronaVac SARS-CoV-2 inactivated vaccine with Huoxiang Suling Shuanghua Decoction: a randomized, double-blind, placebo-controlled study

**DOI:** 10.3389/fimmu.2024.1298471

**Published:** 2024-04-03

**Authors:** Ruying Tang, Linyuan Wang, Jianjun Zhang, Wenting Fei, Rui Zhang, Jinlian Liu, Meiyu Lv, Mengyao Wang, Ruilin Lv, Haipeng Nan, Ran Tao, Yawen Chen, Yan Chen, Yanxin Jiang, Hui Zhang

**Affiliations:** ^1^ School of Chinese Materia Medica, Beijing University of Chinese Medicine, Beijing, China; ^2^ Institute of Chinese Materia Medica, China Academy of Chinese Medical Sciences, Beijing, China; ^3^ School of Traditional Chinese Medicine, Beijing University of Chinese Medicine, Beijing, China

**Keywords:** CoronaVac SARS-CoV-2 inactivated vaccine, Huoxiang Suling Shuanghua Decoction, immunogenicity, clinic trial, metabolomics

## Abstract

**Introduction:**

In light of the public health burden of the COVID-19 pandemic, boosting the safety and immunogenicity of COVID-19 vaccines is of great concern. Numerous Traditional Chinese medicine (TCM) preparations have shown to beneficially modulate immunity. Based on pilot experiments in mice that showed that supplementation with Huoxiang Suling Shuanghua Decoction (HSSD) significantly enhances serum anti-RBD IgG titers after inoculation with recombinant SARS-CoV-2 S-RBD protein, we conducted this randomized, double-blind, placebo-controlled clinical trial aimed to evaluate the potential immunogenicity boosting effect of oral HSSD after a third homologous immunization with Sinovac’s CoronaVac SARS-CoV-2 (CVS) inactivated vaccine.

**Methods:**

A total of 70 participants were randomly assigned (1:1 ratio) to receive a third dose of CVS vaccination and either oral placebo or oral HSSD for 7 days. Safety aspects were assessed by recording local and systemic adverse events, and by blood and urine biochemistry and liver and kidney function tests. Main outcomes evaluated included serum anti-RBD IgG titer, T lymphocyte subsets, serum IgG and IgM levels, complement components (C3 and C4), and serum cytokines (IL-6 and IFN-γ). In addition, metabolomics technology was used to analyze differential metabolite expression after supplementation with HSSD.

**Results:**

Following a third CVS vaccination, significantly increased serum anti-RBD IgG titer, reduced serum IL-6 levels, increased serum IgG, IgM, and C3 and C4 levels, and improved cellular immunity, evidenced by reduce balance deviations in the distribution of lymphocyte subsets, was observed in the HSSD group compared with the placebo group. No serious adverse events were recorded in either group. Serum metabolomics results suggested that the mechanisms by which HSSD boosted the immunogenicity of the CVS vaccine are related to differential regulation of purine metabolism, vitamin B6 metabolism, folate biosynthesis, arginine and proline metabolism, and steroid hormone biosynthesis.

**Conclusion:**

Oral HSSD boosts the immunogenicity of the CVS vaccine in young and adult individuals. This trial provides clinical reference for evaluation of TCM immunomodulators to improve the immune response to COVID-19 vaccines.

## 1 Introduction

The COVID-19 pandemic caused by the SARS-CoV-2 virus has posed a serious threat to human health and life. Vaccination proved to be a safe and effective way to reduce mortality and morbidity from COVID-19. According to the World Health Organization, there were 198 COVID-19 vaccine candidates in preclinical development, 161 COVID-19 vaccine candidates in clinical development, and 11 approved phase IV COVID-19 vaccines as of May 27, 2022 (1). The latter included 1 recombinant protein vaccine, 3 nucleic acid vaccines, 3 inactivated vaccines, and 4 vector vaccines, which have been used in different populations worldwide (2). The CoronaVac SARS-CoV-2 (CVS) inactivated vaccine, developed by Sinovac Life Sciences Co., Ltd. (Beijing, China), is the most widely used COVID-19 vaccine in China. Clinical studies have shown that the vaccine is immunogenic, safe, and effective in the general population (3, 4). Purified, inactivated virus is traditionally used in vaccine development. Inactivated vaccines are composed of intact virus particles rendered non-infectious by treatments that inhibit their ability to infect cells and replicate, and the production process is simple and mature (5). Although inactivated vaccines are generally very safe in all populations, they often produce weak immune responses. Hence, multiple immunizations or combined use of immune enhancers are usually required to boost immunogenicity (6). After vaccination, the presence and persistence of virus-specific serum antibodies are crucial to prevent infection. Results of a phase 2, open-label, randomized controlled trial evaluating a heterologous vaccination schedule indicated that a second vaccination dose with a different COVID-19 vaccine significantly increased virus-specific antibody titers in serum, albeit with a relatively high incidence of mild and moderate adverse effects (7). Therefore, it is of great significance to identify natural, safe, and effective immune enhancers for strengthening and prolonging the immune response induced by COVID-19 vaccines.

In the prevention and control of the epidemic of COVID-19, searching for novel treatments against pathogens is critical. There are hundreds of natural compounds effect on the immunity has performed in preventing and treating the disease’s effects on the respiratory system. Evidences indicated that, colostrum, which contains a higher concentration of conjugated glycans, may be used as a natural alternative to conventional drugs to prevent and/or treat viral diseases such as COVID-19 threatening the health of the general human population (8). Besides, immunomodulatory drugs such as lactoferrin is used to control inflammatory responses in their respective auto-immune conditions, which was demonstrated its effects and mechanisms in the prevention, treatment, and recovery for COVID-19 (9). A clinic trial of lactoferrin suggested that, the COVID-19 patients who were given lactoferrin every day for 10 days, had shown a positive effect and all patients had a faster recovery when compared with the control group (10). Furthermore, traditional Chinese medicine (TCM) was also exerted the prevention and treatment in regulating immunity and inflammation during the outbreak of COVID-19. A randomized controlled trial in exploring the treatment efficacy of TCM Huoxiang Zhengqi dropping pills and Lianhua Qingwen granules on COVID-19 was performed. Results demonstrated that the use of the both TCM combined with western medicine have clinical advantages for COVID-19 patients in improving clinical symptoms, reducing utilization rate of anti-infective drugs, and improving patient prognosis (11). Traditional Chinese medicine has been practiced in China for thousands of years, many of which have immunomodulatory properties, including polysaccharides, saponins, flavonoids, terpenoids and so on (12). Studies have shown that many Chinese medicines can induce dendritic cell maturation and T/B lymphocyte proliferation, activate macrophages, regulate a variety of signaling pathways and cytokines, and have great potential to assist vaccines in exerting innate and adaptive immune responses (13, 14). However, there are few clinical tries were performed to supporting these potential TCM with as vaccine adjuvant. Clinical studies on enhancing the immunogenicity of COVID-19 vaccine with the assistance of traditional Chinese medicine are of great significance to people’s physical and mental health and to provide effective candidate drugs for the prevention and treatment of COVID-19.

Serological detection of virus-directed antibody (IgM, IgG, IgA) titers and neutralizing antibody (NAb) tests are commonly used to evaluate the immunogenic effect of vaccination (15). Usually, in the defense against SARS-CoV-2 infection, the host’s innate immune system is activated to rapidly identify the pathogen. Subsequent activation of adaptive immunity, both humoral (mediated by circulating antibodies produced by B cells), and cellular (mediated mainly by T cells), serves to kill the invading pathogen and protect the body from damage (16). Therefore, enhancing the humoral and cellular immune reserve is an effective strategy to improve the protective effect of vaccine immunization.

Metabolomics uses modern detection techniques combined with bioinformatics analysis methods to perform semi-quantitative or quantitative detection of low molecular weight metabolites in blood, urine, feces, and other biological samples after exposure to pathophysiological stimuli, environmental changes, or drug interventions (17). In a preclinical study, we recently demonstrated that Huoxiang Suling Shuanghua Decoction (HSSD) can treat influenza A virus infection by preventing inflammation and improving immunity (18). Therefore, before conducting the present study, we tested whether HSSD supplementation was able to boost specific serum IgG antibody titers in mice immunized with recombinant SARS-CoV-2 S-RBD protein. Detailed information on methods and results of these experiments are shown in **Supplementary Material 1**, including **Supplementary Figure S1**: Schematic diagram of vaccination and blood sample collection in mouse experiments, **Supplementary Figure S2**: Body weight and serum anti-RBD IgG titers measure after vaccination, **Supplementary Figure S3**: Percentages of CD3^+^, CD4^+^, and CD8^+^ T cells in peripheral mouse blood after vaccination, and **Supplementary Figure S4**: Representative flow cytometry plots of T lymphocyte subsets. As these experiments before proved that HSSD significantly enhanced both humoral and immune cell-based responses to S-RBD inoculation, we conducted a randomized, double-blind, placebo-controlled clinical trial to investigate the effect of oral HSSD supplementation on immunogenicity elicited by a third homologous immunization with the CVS vaccine in adults.

## 2 Materials and methods

A randomized, double-blind, placebo-controlled study design was used in this study. The trial was conducted at Dongfang Hospital, Beijing University of Chinese Medicine, Beijing, China. All study participants (teachers and students) came from the latter institution and were randomly divided (at a 1:1 ratio) into two groups: 1) third CVS vaccine dose + HSSD, and 2) third CVS vaccine dose + placebo, with randomization generated by SPSS Sstatistic’s software (version 20.0). Gender, age, ethnicity, height, body weight, blood pressure, heart rate, electrocardiogram, and interval between the second and third doses of CVS vaccine were recorded to ensure the comparability of the two groups. The study was approved by the Ethics Committee of Beijing University of Chinese Medicine (approval number 2021BZYLL0409). Written informed consent was obtained from each participant. This study was registered with the China Clinical Trial Registration Center (registration number ChiCTR2200055749).

### 2.1 Inclusion and exclusion criteria

Volunteers who met the following criteria were included in this study: (1) 18–60 years old male or female volunteers, without SARS-CoV-2 infection or potential medical conditions; (2) received two doses of CVS vaccine (at a 3–4 weeks interval), but not a third dose; (3) an interval between the second and the third CVS dose of 6–8 months; (4) no serious discomfort within 24 h after the third dose; (5) all three COVID-19 vaccinations were performed with Sinovac’s CVD vaccine; and (6) provided informed consent.

Volunteers who met any one of the following conditions were excluded from the study: (1) a history of SARS-CoV-2 infection; (2) severe adverse reactions such as blood clots and dyspnea within 24 h after receiving the CVS vaccine; (3) severe primary diseases, such as heart, kidney, lung, endocrine, blood, metabolism, or gastrointestinal tract diseases, which may affect the patient’s participation in the trial or the outcome of the study; (4) family history of mental illness or previous mental illness; (5) allergy or multiple drug allergies; (6) pregnant or lactating women.

**Figure 1:**
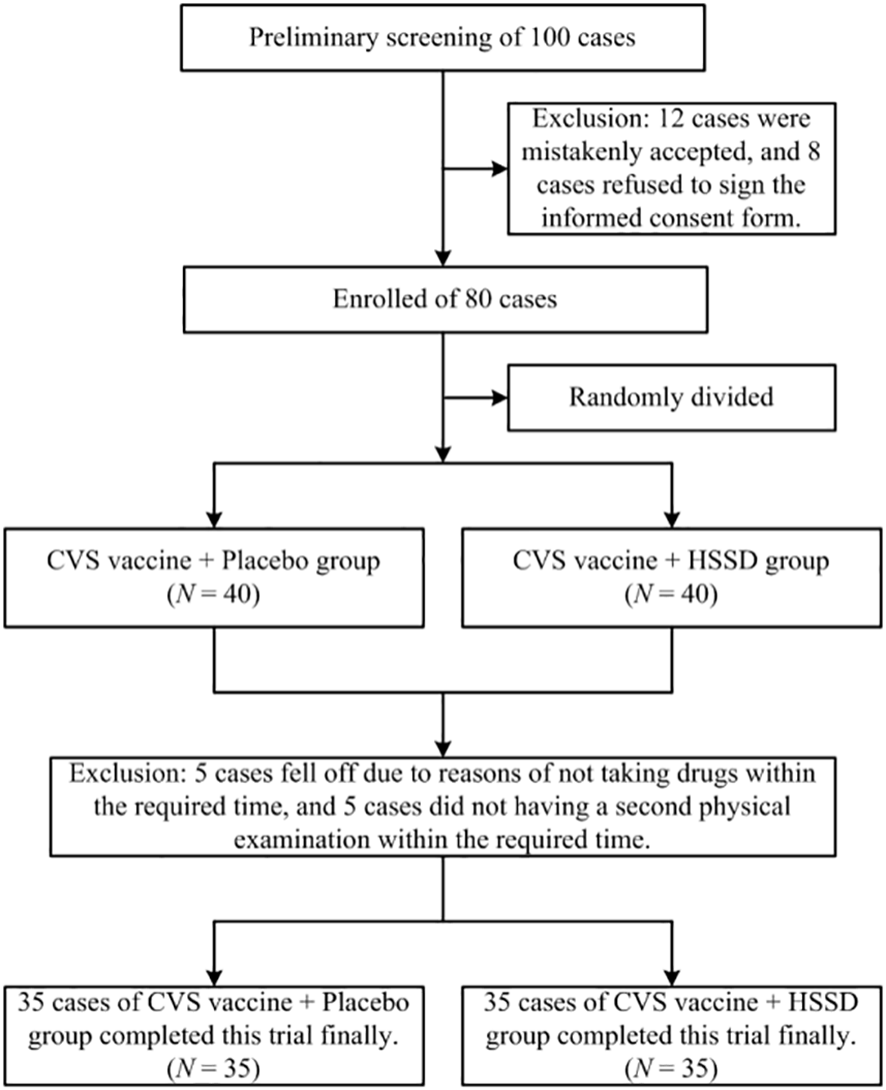
Flowchart of the clinical trial.

In this study, a total of 80 participants were recruited from the Beijing University of Chinese Medicine in Beijing, China from November 1-20, 2021. Aside from the above criteria, participants who met the following criteria were also excluded in the final data analysis: (1) those who were currently taking any drugs or food supplements containing, or interfering with, the HSSD components; (2) those who had participated in another clinical trial in the past 3 months; (3) those who were not willing to comply with the study procedures for any reason. Among the recruited participants, 10 were excluded because of refusal to cooperate during the trial. Finally, a total of 70 subjects completed the study, with a total dropout rate of 12.5% (10/80). A flowchart of this clinical trial is shown in **Figure 1**.

### 2.2 Study medication

The test product Huoxiang Suling Shuanghua Decoction (HSSD, Lot number: 210101, Specification: 6 g in each bag, Tianjin Zhongwei Hezhi Pharmaceutical Co., LTD, Tianjin, China) was a mixture powder containing of 15 kinds of traditional Chinese medicine including their water extract and volatile oil extract. A mixture of 15 kinds of traditional Chinese medicine including Pogostemodis Herba (weight ratio of 1.5), Moslae Herba (weight ratio of 1), Polygonati Rhizoma (weight ratio of 2), Perillae Folium (weight ratio of 1.5), Tsaoko Fructus (weight ratio of 1), Poria (weight ratio of 2), Citri Reticulatae Pericarpium (weight ratio of 1.5), Radix Fici Hirtae (weight ratio of 2), Platycodonis Radix (weight ratio of 1), Zingiberis Rhizoma Recens (weight ratio of 1.5), Jujubae Fructus (weight ratio of 1.5), Lonicerae Japonicae Flos (weight ratio of 0.6), Eriobotryae Folium (weight ratio of 1), Mori Folium (weight ratio of 1), Raphani Semen (weight ratio of 0.5). In our previously study, four compounds of HSSD were identified by the HPLC profile, including chlorogenic acid, caffeic acid, scutellarin, and rosmarinic acid, and the contents of two signature ingredients in the HSSD were determined by HPLC method as chlorogenic acid (0.63 mg/g), and hesperidin (3.13 mg/g), respectively. More details on the ingredients of HSSD were published previously (19). Product standard code of HSSD was GB/T 29602, and its Production License Number was No. SC12712011500236. Besides, product inspection report of HSSD has been comply with relevant regulations by entrusting Tianjin Zhongwei Hezhi Pharmaceutical Co., LTD (Tianjin, China) to execute according to standard of GB/T29602-2013. Furthermore, the toxicological test report of HSSD was also in line with relevant regulations, and Health Food Function Testing Center, College of Applied Arts and Sciences, Peking Union University (Beijing, China) was entrusted to execute according to standards of GB 15193.3-2014, GB 15193.4-2014, GB 15193.5-2014, and GB 15193.8-2014. Also, product inspection report of placebo has been complied with relevant regulations by entrusting Health Food Function Testing Center, College of Applied Arts and Sciences, Peking Union University (Beijing, China) to execute according to standards of GB 5009.3-2016, JJF 1070-2005, GB 4789.2-2016, GB 4789.3-2016, GB 4789.10-2016, GB 4789.15-2016, and GB 4789.4-2016. The nutrients of the HSSD and placebo were shown in **Table 1**. Briefly, HSSD and placebo were manufactured in a good manufacturing practice pilot plant (Tianjin Zhongwei Hezhi Pharmaceutical Co., LTD, Tianjin, China) under quality assurance considering the presence of microorganisms, heavy metals, and pesticide residue. The third-party inspection agency reports indicate that products of HSSD and placebo have quality assurance.

**Table 1:** Nutrients of test products per daily portion.

Nutrients (per 100 g test product)	Placebo	Huoxiang Suling Shuanghua Decoction
Energy, kJ	1444.9	1457
Protein, g	0	0
Fat, g	0	0
Carbohydrate, g	86.1	85.7
Natrium, mg	0	0

### 2.3 Intervention and efficacy evaluation

#### 2.3.1 Research intervention

After randomly allocating study participants into the two aforementioned groups (35 individuals per group), participants in the third CVS vaccine dose + HSSD group and in the third CVS vaccine dose + placebo group started taking HSSD or placebo (sugar, grams/bag), respectively, on the second day after immunization with the CVS vaccine. Participants took one bag of HSSD or placebo twice a day and kept taking them for 7 days. During the trial, participants were banned from taking non-prescription or prescription medications other than HSSD or placebo.

#### 2.3.2 Research efficacy evaluation

All subjects underwent vital signs monitoring and laboratory tests one day before and 14 days after receiving a third dose of the CVS vaccine. At this time, treatment compliance and adverse events (AEs) were also recorded. Clinical and laboratory data were collected regarding blood pressure, heart and respiration rates, routine blood testing (WBC, RBC, HGB, PLT, NEUT%, LYMP%, MON%), routine urine testing (specific gravity, SG; urine pH; urine conductivity, UF; urobilinogen, UBG; urine glucose, GLU; ketone bodies, KET; and urine protein, PRO), electrocardiogram, fasting blood glucose (FBG), liver function (ALT, AST), and kidney function (creatinine, Crea; BUN). In addition, feedback on test products’ compliance and AEs was obtained through a questionnaire on adverse reactions after vaccination.

After collection of peripheral blood, the main outcome measure was serum anti-RBD IgG antibody titer, measured on day 14th after the third vaccine dose. Secondary outcome measures included classification and counting of lymphocyte subsets and serum levels of IgG and IgM, C3 and C4 complement proteins, and IL-6 and IFN-γ, determined both one day before and 14 days after the third vaccine dose. In addition, LC-MS untargeted metabolomics was used to analyze changes in serum analytes indicative of the potential mechanisms by which HSSD may boost CVS vaccine-elicited immunogenicity.

#### 2.3.3 Materials and methods of efficacy evaluation

Serum anti-RBD IgG titers were detected by an ELISA kit (Anti-Novel Coronavirus [2019-nCoV] S-RBD Protein IgG Antibody Detection ELISA kit; Beijing Yiqiao Shenzhou Science and Technology Co., Ltd, China) and calculated as the dilution corresponding to 2.1 times the absorbance value of the negative control, as per the manufacturer’s instructions. According to previous clinical research, seroconversion was defined as a change from seronegative at baseline to seropositive, or a four-fold titer increase if the participant was seropositive at baseline, and the positive cutoff of the titer for anti-RBD IgG was 1:8 (20). ELISA kits for human IgG (IgG, lot number: 20220304A), human IgM (IgM, lot number: 20220304A), human complement protein 3 (C3, lot number: 20220309A), human complement protein 4 (C4, lot number: 20220315A), human interleukin 6 (IL-6, lot number: 20220304A), and human interferon gamma (IFN-γ, lot number: 20220304A) were purchased from Shanghai Enzyme Linked Biotechnology Co., Ltd (Shanghai, China). Whole blood lymphocyte subsets, including T cell (CD3^+^, CD4^+^, and CD8^+^ fractions), B cell, and NK cell populations, were detected by flow cytometry.

### 2.4 Untargeted metabolomics

#### 2.4.1 Metabolite extraction

Serum samples (100 μL) were placed in Eppendorf tubes, resuspended with pre-chilled 80% methanol by vortexing, incubated on ice for 5 min, and centrifuged at 15,000 g, 4°C, for 20 min. A fraction of the supernatant was then diluted in LC-MS grade water to yield a solution containing 53% methanol. The samples were subsequently transferred to fresh Eppendorf tubes and centrifuged again as above. Finally, the supernatants were injected into the LC-MS/MS system analysis.

#### 2.4.2 UHPLC-MS/MS analysis

UHPLC-MS/MS analyses were performed using a Vanquish UHPLC system coupled with an Orbitrap Q Exactive TM HF-X mass spectrometer (Thermo Fisher, Germany). Samples were injected on a Hypersil Gold column (100 × 2.1 mm, 1.9 μm) using a 12 min linear gradient at a flow rate of 0.2 mL/min. The eluents for the positive polarity mode were eluent A (0.1% formic acid in water) and eluent B (methanol). The eluents for the negative polarity mode were eluent A (5 mM ammonium acetate, pH 9.0) and eluent B (methanol). The solvent gradient was set as: 2% B, 1.5 min; 2–85% B, 3 min; 85-100% B, 10 min; 100-2% B, 10.1 min; 2% B, 12 min. The Q Exactive TM HF-X mass spectrometer was operated in positive/negative polarity mode with spray voltage of 3.5 kV, capillary temperature of 320°C, sheath gas flow rate of 35 psi, auxiliary gas flow rate of 10 L/min, S-lens RF level of 60, and auxiliary gas heater temperature of 350°C.

#### 2.4.3 Data processing and metabolite identification

The raw data files generated by UHPLC-MS/MS were inputted into Compound Discoverer 3.1 software (CD3.1, Thermo Fisher) to perform peak alignment, peak picking, and quantitation for each metabolite. The main parameters were set as follows: retention time tolerance, 0.2 min; actual mass tolerance, 5 ppm; signal intensity tolerance, 30%; and signal/noise ratio, 3. After that, peak intensities were normalized to total spectral intensity. Normalized data were used to predict the molecular formula based on additive ions, molecular ion peaks, and fragment ions. The peaks were then matched to the mzCloud (https://www.mzcloud.org/), mzVault (Thermo Fisher), and MassList (http://www.maldi-msi.org/mass) databases to verify metabolite identity and characteristics. Statistical analyses were performed using R software (version R- 3.4.3), Python (version 2.7.6 version) and CentOS (CentOS release 6.6). When data were not normally distributed, normal transformations were attempted using the area normalization method.

#### 2.4.4 Metabolite annotation

The metabolites were annotated using the KEGG database (https://www.genome.jp/kegg/pathway.html), HMDB database (https://hmdb.ca/metabolites), and LIPIDMaps database (http://www.lipidmaps.org/).

### 2.5 Safety monitoring

Toxicological analysis of HSSD (batch number 210101) was performed at the Health Food Function Testing Center of the College of Applied Arts and Sciences of Beijing Union University according to China’s National Food Safety Standards (GB 15193.3-2014: acute oral toxicity test, limited method; GB 15193.4-2014: bacterial reverse mutation test, not using *Escherichia coli*; GB 15193.5-2014: mammalian erythrocyte micronuclear test; and GB 15193.8-2014: chromosome aberration test of mouse spermatogonia or spermatocyte). In addition, good safety was reported for HSSD after a 30-day feeding test in rats.

### 2.6 Statistical analysis

SPSS Statistics 20.0 was used for statistical analysis. Rate and component ratio were represented by *n* (%) and *n/N* (%), respectively. A χ^2^ test was used to compare countable data between groups. When *n* ≥40 and all theoretical frequencies ≥5, Pearson’s χ^2^ statistics was used. When the total frequency *n* was ≥40, but the theoretical frequency was <1, the Fisher’s exact probability test was used. *P <*0.05 was considered significant. Measurement data conforming to normal distribution were expressed as Mean ± Standard Deviation, and t-testing was used for comparison between two groups while one-way analysis of variance (ANOVA) followed by the least significant difference (LSD) *post hoc* test or Dunnett’s T3 test was applied for comparison of multiple groups. Measurement data inconsistent with a normal distribution were expressed as the median (interquartile distance) [*M, (P_25_, P_75_)*], and Mann-Whitney U test was used for comparison between groups. Geometric mean titers (GMT) of RBD IgG antibody were calculated for each group. *P <*0.05 was considered significant. Post-vaccination local and systemic AEs were recorded and classified, and Fisher’s exact probability test subsequently applied to compare differences in incidence and frequencies between groups.

## 3 Results

### 3.1 Clinical characteristics of study subjects

Clinical characteristics of study participants are shown in **Table 2**. Of a total of 100 subjects preliminarily screened, 12 were excluded due to being mistakenly accepted, and another 8 were excluded due to refusal of informed consent. Finally, 80 participants were successfully screened and enrolled. During the study, 5 individuals dropped out for not taking the test substances within the required time, and another 5 were excluded for missing the second physical examination deadline (study dropout rate: 12.5%). A total of 70 subjects (35 in the CVS + placebo group and 35 in the CVS + HSSD group) successfully completed the study. There were 6 males (17%) and 29 females (83%) in each group. Subjects in the CVS + placebo group were aged 18–49 years, while the CVS + HSSD group included 33 individuals aged 18–49 years and 2 individuals aged 50–60 years. In both groups, young people were mainly enrolled. There was no significant difference between the two groups in gender, age, ethnicity, height, body weight, blood pressure, heart rate, electrocardiogram, and interval between the second and third doses of CVS vaccine. All subjects had tested negative for SARS-CoV-2 nucleic acid amplification within three days prior to enrollment.

**Table 2:** Demographic and clinical characteristics of the study participants.

Term	CVS+Placebo group	CVS+HSSD group	χ^2^ or *Z*	*P* value
Gender			0	1
Male	6 (17%)	6 (17%)		
Female	29 (83%)	29 (83%)		
Age, years	21 (20, 22)	25 (24, 30)	-0.739	0.46
Age group				
18 - 49 years	35/35 (100%)	33/35 (94%)		
Male	6/35 (17%)	5/35 (14%)		
Female	29/35 (83%)	28/35 (80%)		
50 - 60 years	0/0 (0%)	2/35 (6%)		
Male	0/0 (0%)	1/35 (3%)		
Female	0/0 (0%)	1/35 (3%)		
Han nationality	31 (89%)	33 (94%)	0.742	0.673
Height (m)	165.0 (160.5, 169.0)	165.5 (159.5, 170.5)	-0.335	0.738
Weight (kg)	58.0 (53.9, 67.8)	58.8 (52.0, 70.0)	-0.035	0.972
Systolic blood pressure (mm Hg)	118 (109, 123)	119 (109, 122)	-0.878	0.380
Diastolic blood pressure (mm Hg)	73 (68, 78)	73 (66, 78)	-0.535	0.592
Abnormal heart rate cases	0 (0%)	0 (0%)	–	–
Abnormal electrocardiogram cases	0 (0%)	0 (0%)	–	–
Time between the second and the third dose of CVS (Weeks)	30 (28, 32)	29 (28, 31)	-0.143	0.886
Negative nucleic acid proof	35 (100%)	35 (100%)	–	–

Data were presented as *M* (*P_25_, P_75_*), *n* (%), or *n/N* (%) unless otherwise specified, *N* = 35.

### 3.2 HSSD boosts the immunogenicity of the CoronaVac SARS-CoV-2 vaccine

#### 3.2.1 Analysis of serum anti-RBD IgG titer positivity rate and titer range

As shown in **Table 3**, one day before receiving the third dose of the CVS vaccine (baseline), in all study participants the serum anti-RBD IgG titer positivity rate was 100%. Serum anti-RBD IgG antibody titers ranging from 1:80 to 1:160 were observed in 69% of participants allocated to the CVS + placebo group and in 63% of participants in the CVS + HSSD group. Meanwhile, on day 14 after the third CVS dose, serum anti-RBD IgG positivity rate was 60% in the CVS + Placebo group and 92% in the CVS + HSSD group (χ^2^ = 9.401, *P* < 0.01). At this time point, serum anti-RBD IgG antibody titers ranging from 1:400 to 1:1600 were recorded in 74% of subjects in the CVS + placebo group and in 69% of subjects in the CVS + HSSD group. Confirming HSSD’s boosting immunogenic effect, after the third CVS vaccine dose significantly increased anti-RBD IgG antibody titers, evidenced by a 10-fold vs 5-fold increase in the GMT ratio (estimated by ratioing median GMT values observed after and before vaccination) were recorded in the CVS + HSSD group relative to the CVS+ placebo group (*Z* = −3.26, *P* < 0.01).

**Table 3:** Changes of clinic serum anti-RBD IgG titer positive rate, positive turn rate, and titer range.

Term	CVS + Placebo group		CVS + HSSD group		χ^2^ or *Z*	*P* value
	Cases	*n/N* (%)	Cases	*n/N* (%)		
One day before the third dose of CVS vaccine						
Serum antibody positive rate	35	100%	35	100%		
Antibody titer < 8	0	0%	0	0%		
Antibody titer = 8 ~ 40	6	17%	9	26%		
Antibody titer = 80 ~ 160	24	69%	22	63%		
Antibody titer ≥ 320	5	14%	4	11%		
On day 14 after the third dose of CVS vaccine						
Serum antibody positive turn rate	21	60%	32	92%^**^	9.401	0. 0020
Antibody titer < 8	0	0%	0	0%		
Antibody titer = 8 ~ 200	6	17%	3	8%		
Antibody titer = 400 ~ 1600	26	74%	24	69%		
Antibody titer ≥ 3200	3	9%	8	23%		
The multiple of GMT after the third dose of CVS vaccine divided by GMT before the dose [*M (P_25_, P_75_)*]	5 (2.5, 5)		10 (5, 20)^**^		- 3.26	0. 0011

Data were presented as *n/N* (%) or *M* (*P*_25_, *P*_75_), *N* = 35, **P < 0.01: significantly differently from the CVS + Placebo group.

#### 3.2.2 Changes in serum anti-RBD IgG antibody titers

**Figure 2:**
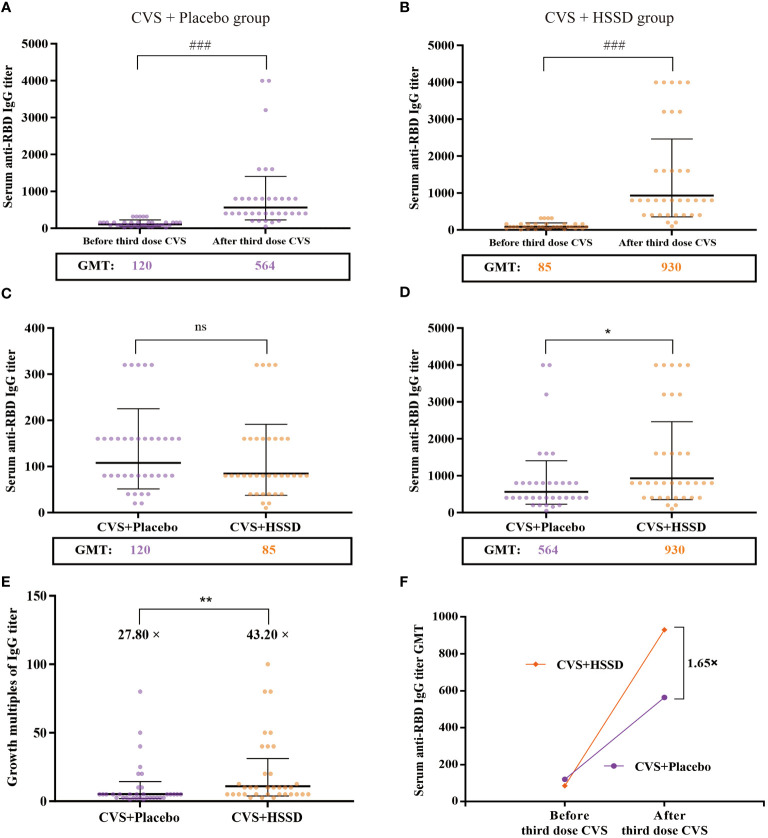
Changes of clinic serum anti-RBD IgG antibody titer and its GMT. **(A)** Serum anti-RBD IgG antibody titer of CVS + Placebo group, **(B)** Serum anti-RBD IgG antibody titer of CVS + HSSD group, **(C)** Serum anti-RBD IgG antibody titer of one day before the third dose of CVS vaccine, **(D)** Serum anti-RBD IgG antibody titer on day 14 after the third dose of CVS vaccine, **(E)** Growth multiples of serum anti-RBD IgG antibody titer, **(F)** Growth multiples of serum anti-RBD IgG antibody titer GMT. Data were presented as Geometric Mean ± Geometric SD, N = 35, ###P < 0.001: significantly differently from one day before the third dose of CVS vaccine, *P < 0.05, **P < 0.01: significantly differently from the CVS + Placebo group.

As shown in **Table 4** (longitudinal view), and **Figures 2A, B**, in both study groups serum anti-RBD IgG titer was significantly increased on the 14th day after the third dose of CVS vaccine, compared with values recorded one day before its application (CVS + placebo group: *Z* = −6.556, *P* < 0.001; CVS + HSSD group: *Z* = −7.017, *P* < 0.001). As shown in **Table 4** (horizontal view), and **Figure 2C**, there was no significant difference among groups in serum in anti-RBD IgG titer at one day before the third CVS vaccine dose (*Z* = −1.315, *P* > 0.05), with a GMT of 120 in the CVS + placebo group and of 85 in the CVS + HSSD group. In contrast, as shown in **Table 4** (horizontal view), and **Figure 2D**, on the 14th day after the third CVS vaccine dose serum anti-RBD IgG titers were significantly increased in participants supplemented with HSSD (*Z* = −2.183, *P* < 0.05). In **Figure 2E**, the corresponding GMTs (564 in the CVS + placebo group and 930 in the CVS + HSSD groups) represented, respectively, a 27.8-fold and a 43.2-fold GMT increase (*P* < 0.01). As shown in **Figure 2F**, on day 14 after the third dose of CVS vaccine, GMT in the CVS + HSSD group was 1.65 times (930/564) higher than that in the CVS + placebo group. These findings indicate that HSSD can significantly boost the increase in serum IgG specific for RBD after a third CVS vaccine dose.

**Table 4:** Changes of clinic serum anti-RBD IgG antibody titer.

Term	CVS + Placebo group	CVS + HSSD group	*Z* value	*P* value
One day before the third dose of CVS vaccine [titer, *M (P_25_, P_75_)*]	160 (80, 160) GMT = 120	80 (40, 160) GMT = 85	- 1.315	0.189
On day 14 after the third dose of CVS vaccine [titer, *M (P_25_, P_75_)*]	400 (400, 800) GMT = 564	800 (400, 16000) ^###*^ GMT = 930	- 2.183	0.029
*Z* value	- 6.556	- 7.017	–	–
*P* value	< 0.001	< 0.001	–	–

Data were presented as *M* (*P*_25_, *P*_75_), *N* = 35, ###P < 0.001: significantly differently from one day before the third dose of CVS vaccine, *P < 0.05: significantly differently from the CVS + Placebo group.

#### 3.2.3 Receiver operating characteristic of serum anti-RBD IgG titer

**Figure 3:**
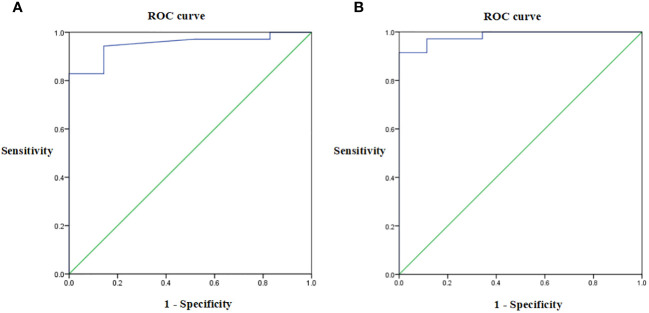
Receiver operating characteristic of serum anti-RBD IgG titer in **(A)** CVS + Placebo group, **(B)** CVS + HSSD group, respectively. *N* = 35.

ELISA was used to detect serum anti-RBD IgG antibody levels. As shown in **Figure 3**; **Table 5**, for both study groups an optimal cut-off GMT value of 360 was determined for serum anti-RBD IgG titer before and after the third CVS vaccine dose, according to receiver operating characteristic (ROC) curve analysis. The area under the ROC curve (ROC AUC) was 0.951 in the CVS + placebo group and 0.984 in the CVS + HSSD group. These results showed that at the above cut-off value, there was good detection performance for serum anti-RBD IgG levels in subjects from both groups. These data indicated that ELISA had good sensitivity and strong specificity, hence titration of RBD IgG antibodies by this method was reliable.

**Table 5:** Receiver operating characteristic information.

Groups	AUC	95% CI	Cutoff value	Sensitivity	Specificity	Maximum Jorden index
CVS + Placebo group	0.951	0.897 – 1.000	360	0.829	1	0.829
CVS + HSSD group	0.984	0.960 – 1.000	360	0.914	1	0.914

### 3.3 HSSD improves immunoglobulin, cytokine, and lymphocyte distribution profiles after third vaccination with CVS

#### 3.3.1 Changes in serum immunoglobulins and cytokines

As shown in **Table 6** (longitudinal view), compared with baseline, in both study groups the levels of serum IgG, IgM, and complement (C3 and C4) proteins were significantly increased on the 14^th^ day after the third CVS vaccine dose (*P* < 0.001). In turn, serum IL-6 levels were significantly decreased (*P* < 0.001), while those of IFN-γ showed no change (*P* > 0.05). These results showed that vaccination with CVS significantly increases serum IgG, IgM, C3, and C4 levels while reducing those of pro-inflammatory IL-6. As shown in **Table 6** (horizontal view), there were at baseline no significant differences in serum levels of IgG, IgM, C3, C4, IL-6, and IFN-γ among groups (*P* > 0.05). In contrast, on the 14^th^ day after the third CVS dose, in the HSSD group the levels of IgG, IgM, C3, and C4 were significantly increased (*P* < 0.05, *P* < 0.01), IL-6 levels were significantly decreased (*P* < 0.001), and no significant difference was detected in IFN-γ (P > 0.05) relative to the CVS+ placebo group. These results further suggest that HSSD potentiates the immunogenic effect of the CVS vaccine and counteracts also inflammation by further reducing serum IL-6.

**Table 6:** Changes of clinic serum immunoglobulins and cytokines before and after third dose of CVS vaccine.

Term	CVS + Placebo group	CVS + HSSD group	*Z* value	*P* value
0 day: Immunoglobulin IgG (mg/mL)	16.32 (12.85, 18.85)	14.86 (12.75, 17.94)	1.075	0.282
14 days: Immunoglobulin IgG (mg/mL)	23.74 (21.45, 26.35) ^###^	25.44 (23, 28.23) ^###*^	2.038	0.042
*Z* value	- 7.007	- 7.195	–	–
*P* value	< 0.001	< 0.001	–	–
0 day: Immunoglobulin IgM (μg/mL)	1140.53 (846.79, 1309.54)	1165.98 (931.81, 1248.45)	0	1
14 days: Immunoglobulin IgM (μg/mL)	1572.72 (1429.98, 1697.97) ^###^	1723.63 (1528.5, 1922.12) ^###**^	2.614	0.009
*Z* value	- 6.725	- 7.101	–	–
*P* value	< 0.001	< 0.001	–	–
0 day: complement C3 (μg/mL)	564.98 (473.62, 637.54)	545.71 (467.67, 643.96)	0.264	0.792
14 days: complement C3 (μg/mL)	879.96 (786, 985.58) ^###^	985.32 (850.71, 1026.78) ^###*^	1.991	0.046
*Z* value	- 7.018	- 7.171	–	–
*P* value	< 0.001	< 0.001	–	–
0 day: complement C4 (μg/mL)	226.22 (178.34, 285.79)	220.56 (176.72, 259.53)	0.487	0.626
14 days: complement C4 (μg/mL)	359.36 (326.98, 412.21) ^###^	416.57 (360.74, 439.99) ^###*^	2.555	0.011
*Z* value	- 7.195	- 7.148	–	–
*P* value	< 0.001	< 0.001	–	–
0 day: IL-6 (pg/mL)	45.68 (39.57, 50.91)	42.31 (39.15, 46.73)	1.498	0.134
14 days: IL-6 (pg/mL)	28.35 (24.95, 31.72) ^###^	23.18 (19.75, 26.32) ^###***^	4.035	< 0.001
*Z* value	- 7.159	- 7.147	–	–
*P* value	< 0.001	< 0.001	–	–
0 day: IFN-γ (pg/mL)	486.96 (391.48, 556.95)	465.79 (383.71, 537.51)	0.352	0.725
14 days: IFN-γ (pg/mL)	419.78 (366.96, 537.08)	472.06 (393.9, 573.83)	1.057	0.29
*Z* value	- 1.18	- 0.17	–	–
*P* value	0.238	0.865	–	–

0 day: one day before the third dose of CVS vaccine, 14 days: on day 14 after the third dose of CVS vaccine. Data were presented as *M* (*P*_25_, *P*_75_), *N* = 35, ###*P* < 0.001: significantly differently from one day before the third dose of CVS vaccine, *P < 0.05, **P < 0.01, ***P < 0.001: significantly differently from the CVS + Placebo group.

#### 3.3.2 Changes in lymphocyte subset distribution

As shown in **Table 7** (longitudinal view), compared with the day before the third CVS dose, by day 14 post-immunization the frequency of CD3^+^CD4^+^ and CD3^+^CD8^+^ cells in the HSSD group were significantly reduced (*P* < 0.05). In turn, as shown in **Table 7** (horizontal view), compared with the placebo group, on the 14^th^ day after the third CVS dose the lymphocyte absolute count, CD3^+^, CD3^+^CD4^+^, and CD3^+^CD8^+^ frequencies, and the CD4^+^/CD8^+^ ratio was all significantly reduced in the HSSD group (*P* < 0.05). These results showed that HSSD beneficially regulates T cell subset distribution, thus helping balancing immune function after vaccination with CVS.

**Table 7:** Changes of clinic lymphocyte subsets before and after third dose of CVS vaccine.

Term	CVS + Placebo group	CVS + HSSD group	χ^2^ value	*P* value
0 day: abnormal cases of total lymphocyte absolute count (μL)	7 (20%)	5 (14%)	0.402	0.526
14 days: abnormal cases of total lymphocyte absolute count (μL)	6 (17%)	0 (0%) ^*^	–	0.025
χ^2^ value	0.094	–	–	–
*P* value	0.759	0.054	–	–
0 day: abnormal cases of CD3^+^ % (%)	4 (11%)	4 (11%)	–	1
14 days: abnormal cases of CD3^+^ % (%)	8 (23%)	1 (3%) ^*^	–	0.028
χ^2^ value	1.609	–	–	–
*P* value	0.205	0.356	–	–
0 day: abnormal cases of CD3^+^CD4^+^ % (%)	9 (26%)	10 (29%)	0. 072	0.788
14 days: abnormal cases of CD3^+^CD4^+^ % (%)	8 (23%)	2 (6%) ^#*^	4.200	0.04
χ^2^ value	0. 078	6.437	–	–
*P* value	0.78	0.011	–	–
0 day: abnormal cases of CD3^+^CD8^+^ % (%)	4 (11%)	6 (17%)	0. 467	0.495
14 days: abnormal cases of CD3^+^CD8^+^ % (%)	6 (17%)	0 (0%) ^#*^	–	0.025
χ^2^ value	0. 467	–	–	–
*P* value	0.495	0.025	–	–
0 day: abnormal cases of CD4^+^/CD8^+^ ratio	7 (20%)	7 (20%)	0	1
14 days: abnormal cases of CD4^+^/CD8^+^ ratio	8 (23%)	1 (3%) ^*^	6.248	0.012
χ^2^ value	0.085	–	–	–
*P* value	0.771	0.055	–	–
0 day: abnormal cases of CD19^+^ % (%)	3 (9%)	2 (6%)	–	1
14 days: abnormal cases of CD19^+^ % (%)	6 (17%)	2 (6%)	–	0.259
χ^2^ value	–	–	–	–
*P* value	0.477	1	–	–
0 day: abnormal cases of CD16^+^CD56^+^ % (%)	3 (9%)	3 (9%)	–	1
14 days: abnormal cases of CD16^+^CD56^+^ % (%)	6 (17%)	4 (11%)	0. 467	0.495
χ^2^ value	–	–	–	–
*P* value	0.477	1	–	–

0 day: one day before the third dose of CVS vaccine, 14 days: on day 14 after the third dose of CVS vaccine. Data were presented as n (%), *N* = 35, #*P* < 0.05: significantly differently from one day before the third dose of CVS vaccine, *P < 0.05: significantly differently from the CVS + Placebo group. The normal range values of each index were as follows, and the cases that did not fall within the corresponding range was considered as the abnormal cases: Normal range of total lymphocyte absolute count: 1530 - 3700 μL, Normal range of CD3^+^ %: 50 % - 84 %, Normal range of CD3^+^CD4+ %: 27 % - 51 %, Normal range of CD3^+^CD8^+^ %: 15 % - 44 %, Normal range of CD4+/CD8+ ratio: 0.71-2.78, Normal range of CD19^+^ %: 5 % - 18 %, Normal range of CD16^+^CD56^+^ %: 7 % - 40 %.

### 3.4 Untargeted serum metabolomics analysis

#### 3.4.1 Quality assessment, quality control, principal component analysis, and orthogonal partial least squares discriminant analysis of serum metabolite data

**Figure 4:**
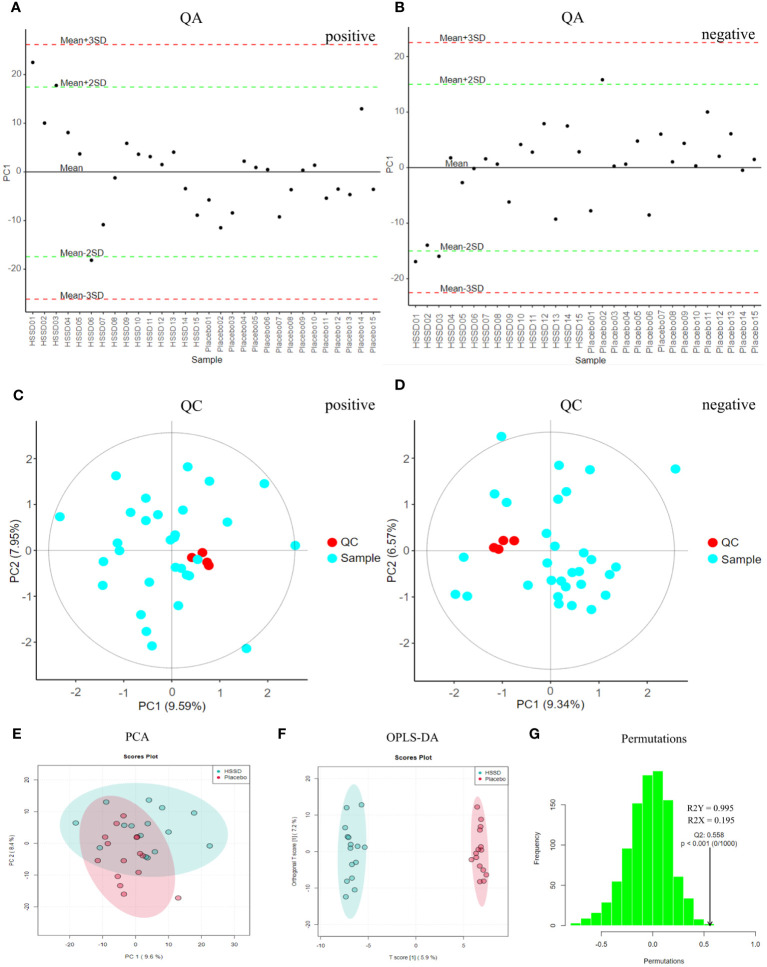
QA, QC, PCA, and OPLS-DA results of day 14 after third dose of CVS vaccine serum samples. **(A)** QA results of positive mode, **(B)** QA results of negative mode, **(C)** QC results of positive mode, **(D)** QC results of negative mode, **(E)** PCA results, **(F)** OPLS-DA results, **(G)** Permutation test results of OPLS-DA.

As shown in **Figures 4A, B**, loading plots generated from metabolomics data obtained 14 days after the third CVS dose showed that most of the points fluctuated around the mean axis, within 2 SD from the mean, and no sample point exceeded the control limit by 3 SD. This indicated that there were no outliers in the samples, and that sample preparation and measurement procedures were controllable. The QC-based random forest signal correction (QC-RFSC) algorithm of the stat Target package in R language was used to correct characteristic signal peaks in each serum sample, and the correction effect of each metabolite was recorded. As depicted in **Figures 4C, D**, the red QC sample points clustered together in the PCA plot after signal drift correction, which proved that the correction effect was good. Meanwhile, unsupervised PCA results (shown in **Figure 4E**) showed that there were some data clouds in different regions, suggesting differences in metabolite composition between the two groups. The latter was further verified upon OPLS-DA modeling, which revealed differential distribution of data clouds in samples from each group (**Figure 4F**). In permutation tests on OPLS-DA models (R2X = 0.195, R2Y = 0.995, Q2 = 0.558; **Figure 4G**), the actual observed Q2 indicated by the arrow is on the right side of the random distribution (the observed value is obviously higher than the random value), indicating that Q2 is significant, not random, and the predictive ability of the model is significant. These data thus confirmed that on the 14^th^ day after the third CVS dose there were differentially regulated metabolites between the CVS + placebo and the CVS + HSSD groups.

#### 3.4.2 Screening and identification of differential metabolites in serum samples

**Figure 5:**
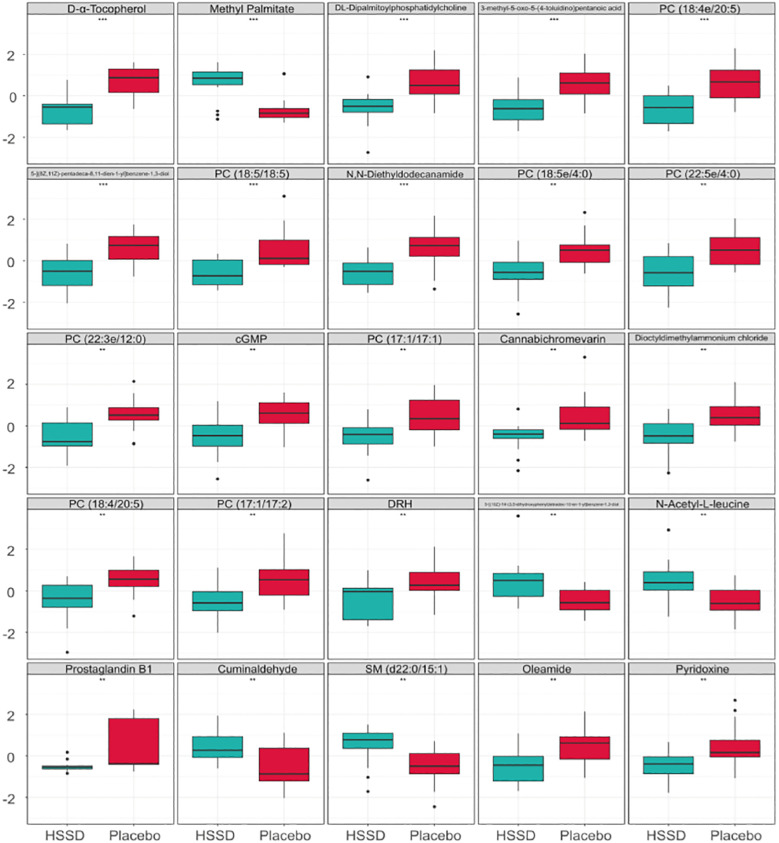
Differential metabolites box plot of serum samples on day 14 after third dose of CVS vaccine *n* = 15, **P < 0.01, ***P < 0.001: significantly differently from the CVS + Placebo group.

The differential metabolites detected in serum samples of subjects on day 14 after the third CVS dose were screened based on VIP > 1 in the OPLS-DA model and *P <*0.05 in *t*-test. Then, a total of 99 differential serum metabolites that contributed greatly to discriminating the CVS + placebo from the CVS + HSSD group were screened. A box plot of the top 25 differentially regulated (P-value based) metabolites thus detected was obtained by single-dimensional statistical analysis (**Figure 5**). These included D-α tocopherol, methyl palmitate, DL- dipalmitoyl phosphatidylcholine, 3-methyl-5-oxo-5-(4-toluidino)-pentanoic acid, 5-[(8Z,11Z)-pentadeca-8,11-dien-1-yl]benzene-1,3-diol, PC (18:5/18:5), N,N-diethyldodecanamide, PC (18:5e/4:0), PC (22:5e/4:0), PC (22:3e/12:0), cGMP, PC (17:1/17:1), cannabichromevarin, dioctyldimethylammonium chloride, PC (18:4/20:5), PC (17:1/17:2), 5-[(10Z)-14-(3,5-dihydroxyphenyl)tetradec-10-en-1-yl]benzene-1,3-diol, N-acetyl-L-leucine, prostaglandin B1, cuminaldehyde, oleamide, and pyridoxine. Among those, 5 metabolites, including methyl palmitate, 5-[(10Z)-14-(3,5-dihydroxyphenyl) tetradec-10-en-1-yl] benzene-1,3-diol, N-acetyl-L-leucine, cuminaldehyde, and SM (d22:0/15:1) were upregulated in the serum of subjects in the HSSD group. **Table 8** shows, for both study groups, the top 15 metabolites classified by support vector machines (SVM) analysis. These included D-α-tocopherol, DL-dipalmitoyl phosphatidylcholine, methyl palmitate, prostaglandin B1, cannabichromevarin, PC (22:3e/12:0), 5-[(8Z,11Z)-pentadeca-8,11-dien-1-yl]benzene-1,3-diol, PC (18:4e/20:5), SM (d22:0/15:1), DRH, PC(18:5e/18:5), androstenedione, 3-(3,4,5-trimethoxyphenyl)propanoic acid, PC (18:5/18:5), and pyridoxine. Based on the above analyses, significant increases in methyl palmitate, SM (d22:0/15:1), PC (18:5e/18:5), and 3-(3,4,5-trimethoxyphenyl) propanoic acid were observed on day 14 after the third CVS dose in subjects supplemented with HSSD.

**Table 8:** Results information of support vector machine screening of serum differential metabolites of subjects on day 14 after the third dose of CVS vaccine.

Number	Differential metabolites name	m/z	Rt (min)	*P* value	Average Importance	CVS + HSSD group/CVS + Placebo group
1	D-α-Tocopherol	431.39	7.43	0.000018205	0.00012968	↓
2	DL-Dipalmitoyl phosphatidylcholine	734.57	9.69	0.00043851	0.00012714	↓
3	Methyl Palmitate	288.29	6.32	0.000141232	0.00024700	↑
4	Prostaglandin B1	337.24	8.75	0.000138572	0.00627940	↓
5	Cannabichromevarin	287.20	7.50	0.0034099	0.00014030	↓
6	PC (22:3e/12:0)	742.58	9.29	0.0016597	0.00012511	↓
7	5 - [(8Z, 11Z) - pentadeca - 8,11 - dien - 1 - yl] benzene - 1,3 - diol	317.25	10.20	0.00055338	0.00010506	↓
8	PC (18:4e/20:5)	786.53	10.71	0.0054521	0.00005076	↓
9	SM (d22:0/15:1)	745.62	11.73	0.0066218	0.00007011	↑
10	DRH	449.19	6.15	0.0056922	0.00011248	↓
11	PC (18:5e/18:5)	756.50	7.96	0.011198	0.00010477	↑
12	Androstenedione	287.20	6.57	0.014025	0.00011126	↓
13	3-(3, 4, 5-trimethoxyphenyl) propanoic acid	263.09	6.04	0.011797	0.00009610	↑
14	PC (18:5/18:5)	770.47	9.52	0.00073134	0.00009745	↓
15	Pyridoxine	170.08	10.79	0.0072871	0.00008755	↓

"↑" means the levels of metabolites in CVS + HSSD group higher than that in CVS + Placebo group, while "↓" means the levels of metabolites in CVS + HSSD group lower than that in CVS + Placebo group.

#### 3.4.3 Metabolic pathway enrichment analysis

As shown in **Table 9**, the metabolic pathways significantly enriched in metabolites differentially regulated by HSSD were mainly concentrated in purine metabolism, vitamin B6 metabolism, folate biosynthesis, arginine and proline metabolism, and steroid hormone biosynthesis, which suggested their potential mechanistic involvement in the boosting effect of HSSD on immunogenicity elicited by the CVS vaccine.

**Table 9:** Enriched pathway information of differential metabolites.

KEGG	KEGG pathway name	*P* value	FDR	Impact
hsa00230	Purine metabolism	0.085755	1	0.02143
hsa00750	Vitamin B6 metabolism	0.16006	1	0.075
hsa00790	Folate biosynthesis	0.205	1	0.01887
hsa00330	Arginine and proline metabolism	0.34541	1	0.00971
hsa00140	Steroid hormone biosynthesis	0.42154	1	0.05085

### 3.5 Safety assessment

#### 3.5.1 Adverse events following third dose CVS vaccination

**Figure 6:**
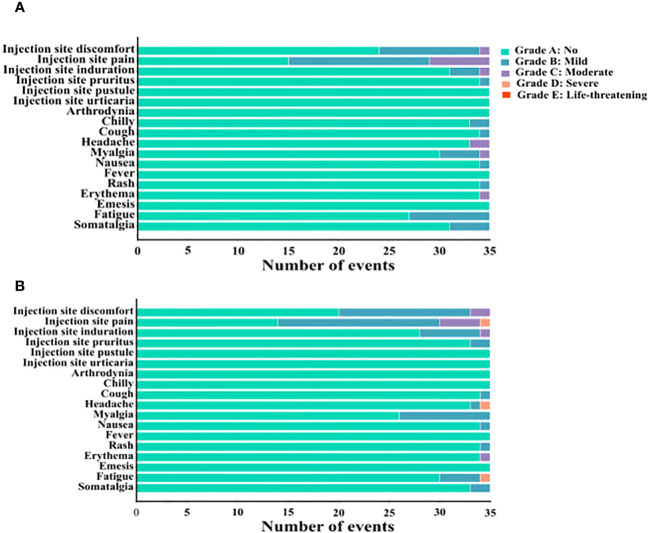
The number of adverse events occurred on the day of the third CVS vaccination in **(A)** CVS + Placebo group, and **(B)** CVS + HSSD group.

AEs occurring after the third CVS vaccine dose were analyzed by questionnaire. Evidence of local AEs at the injection site included discomfort, pain, induration, pruritus, pustule, and urticaria. Systemic AEs evaluated included arthrodynia, chills, cough, headache, myalgia, nausea, fever, rash, erythema, emesis, fatigue, and somatalgia. The above reactions were graded according to their severity: Grade A: No symptoms; Grade B: Mild; Grade C: Moderate; Grade D: Severe; Grade E: Life-threatening. As shown in **Figure 6**, on the day of the third CVS vaccination, the incidence of local AEs in the CVS + placebo and CVS + HSSD groups was comparable (17% vs 21%, respectively; χ^2^ = 0.520, *P* > 0.05). The severity of local reactions was mainly grading B, and their incidence did not differ among groups (13% vs 18%, respectively; χ^2 ^= 0.954, *P* > 0.05). The incidence of local reactions reaching grade C was 4% and 3% respectively (*P* = 1, Fisher’s exact test), and the number of patients with local reactions reaching grade D was 0 and 1, respectively (*P* = 1, Fisher’s exact test). No AEs of grade E severity were observed.

On the day of the third CVS vaccination, the incidence of systemic AEs in the CVS + placebo group and the CVS + HSSD group was 6% and 5%, respectively (χ^2^ = 0.096, *P* > 0.05). The severity of systemic reactions was mainly grading B, with identical incidence (5%) in both groups (χ^2^ = 0, *P* > 0.05). The incidence of systemic reactions reaching grade C was 1% and 0%, respectively (*P* = 1, Fisher’s exact test). The number of patients with systemic reactions reaching grade D was 0 and 2, respectively (*P* = 0.497, Fisher’s exact test). No AEs of grade E severity were observed.

#### 3.5.2 Examination of liver function, renal function, fasting blood-glucose, and routine blood and urine analyses

Compared with one day before the third CVS dose, by day 14 post-immunization no significant differences in safety indexes of liver function (ALT, AST), renal function (Crea, BUN), FBG, nor routine blood (WBC, RBC, HGB, PLT, NEUT%, LYMP%, MON%) and urine (SG, pH, UF, UBG, GLU, KET, and PRO) screening were detected among the CVS + placebo and the CVS + HSSD groups (all *P* > 0.05). Values for the above parameters were all within normal reference ranges for healthy people, indicating that the third CVS vaccine dose was safe. Likewise, compared with the CVS + placebo group, no significant differences in the above safety indexes were recorded in the CVS + HSSD group (*P* > 0.05), both at baseline and on day 14 after the third CVS dose. These data indicated that oral administration of HSSD was safe. Detailed information on biochemical test results is provided in **Supplementary Material 2** of **Supplementary Table S1**: Examination results of liver function, renal function, fasting blood-glucose, blood routine, and routine urine before and after third dose of CVS vaccine in all subjects.

## 4 Discussion

Inactivated virus and recombinant protein-based vaccines approved for clinical use are considered very safe. However, in most people their immunogenic effect is relatively low, which requires multiple vaccination doses or combined use of immune enhancers or vaccine adjuvants (21, 22). Adjuvants are indispensable components of vaccines, required to elicit protective immune responses to non-replicating, inactivated, and subunit antigens (23). Aluminum hydroxide is the only vaccine adjuvant approved for clinical use in China, and has been applied in the production of protein subunit vaccines against COVID-19, hepatitis B, diphtheria, and tetanus (24). The study of the immune effects of traditional Chinese medicine (TCM) compounds is of great interest. Several herbal tonics used in TCM have shown to enhance immunity with little or no toxicity or side effects, and may potentially be used as vaccine adjuvants to promote the secretion of immune-activating factors, activate B cells and T cells, and enhance the body’s humoral and cellular immunity (25). Our previous research showed that HSSD may aid the treatment of influenza A virus infection by eliciting anti-inflammatory and immunoregulatory effects (18). Our proof-of-concept study in mice immunized with SARS-CoV-2 S-RBD recombinant protein showed that HSSD supplementation significantly enhanced serum anti-RBD IgG titers, increased the percentage of CD4^+^ T cells and boosted the secretion of related cytokines, and regulated adaptive immunity. These preclinical results initially suggested that HSSD may improve antibody titer response to SARS-CoV-2 vaccines, laying the foundation for this clinical trial.

The results of this study showed that HSSD enhances the seroconversion rate after vaccination with a third CVS dose, significantly improving its protective effect. Clinical evaluation of specific serum antibody titers (IgM, IgG, IgA) and neutralizing antibody (NAb) levels is used to evaluate immunogenicity after vaccination against SARS-CoV-2 and other pathogens (26, 27). In contrast, positive conversion to NAb was not seen until 4 weeks after the first dose (28). Therefore, according to the 2-week duration of this clinical trial, analysis of serum specific anti-RBD IgG titers was selected to evaluate the efficacy of HSSD in boosting the immunogenic effect of a third dose of inactivated CVS vaccine. Research demonstrated that the antibodies produced by the inactivated vaccine are still detectable in humans up to 6 months after the second dose. However, its effectiveness is variously affected by factors such as underlying diseases, age, gender, and interval between initial a booster doses (29, 30). Previous studies have shown that the seroconversion rate of antibodies against SARS-CoV-2 decreases by nearly 50% at 90 days after administration of two doses of CVS vaccine, with further decrease noted by 180 days. Since the remaining levels of serum antibodies in seropositive individuals are too low to provide effective protection against infection, a third dose of CVS vaccine immunization becomes necessary (31). Therefore, in this trial the interference of gender, age, nationality, height, weight, blood pressure, heart rate, electrocardiogram, interval between the second and third dose of CVS vaccination, and other confounding factors was excluded to ensure that baseline characteristics were comparable among the two study groups.

Previous research has shown that inactivated COVID-19 vaccines tend to produce weak cellular immunity and mainly induce humoral responses through the MHC II pathway (32). As part of the adaptive immune response, humoral immunity is an important body defense system that effectively monitors and prevents virus infection (33). The levels of serum IgG, IgM, and complement C3 and C4 are the main indicators reflecting humoral immunity (34, 35). Research has demonstrated that serum IgG antibodies neutralize toxins and thus reduce infection risk by activating complement and enhancing the phagocytosis of pathogens (36). IgM is the first antibody isotype produced in response to initial antigen exposure, and thus plays a critical role in host protection (37, 38). Activation of the complement system (usually evaluated by measurement of serum C3 and C4 levels) contributes to the body’s immune response by activating inflammatory cells and promoting pathogen opsonization and lysis (39). In addition, studies have shown that T cells play a key role in maintaining long-term vaccine immunity to against COVID-19 infection. The two main T cell populations include CD8^+^ and CD4^+^ T cells, both of which are primed by antigen and costimulatory signals (40). When activated, naïve T cells can rapidly proliferate and differentiate into CD4^+^ helper T cells, cytotoxic CD8^+^ T cells, and regulatory T cells and adopt effector, memory, or regulatory functions. Memory CD4^+^ T cells induce B cells to produce antibodies to resist reinfection with the same antigen, thereby triggering an antigen-specific immune response (41, 42). Therefore, CD4^+^ T cell and CD8^+^ T cell responses represent useful indicators of the immune effect of vaccines, and the dynamic balance of the CD4^+^/CD8^+^ ratio is considered a significant marker of immune status (43). IFN-γ is an important immunomodulatory and antiviral cytokine, involved in the activation of cellular immunity. IL-6 is a potent proinflammatory cytokine closely involved in the hyperinflammatory state (cytokine storm) associated with severe COVID-19 infection, and its inhibition may reduce the severity of the disease (44). Results of this trial demonstrated that oral HSSD can enhance the immunogenic effect of the CVS vaccine by promoting the production of IgG, IgM, and complement C3 and C4 related to humoral immunity, balancing cellular immunity, and reducing balance deviations in distribution and counts of lymphocyte subsets, which may be related to regulation of activation, proliferation, survival, differentiation, and migration of T cells. Therefore, oral supplementation with HSSD proved to effectively boost the immunogenicity of a third immunization with the CVS vaccine by enhancing humoral and cellular immune responses and reducing IL-6-dependent respiratory inflammation.

Maintaining the homeostasis and health of the immune system is critical for preventing infection. In this trial, after a third CVS dose 15 representative differential metabolites were identified by serum non-targeted metabolomics in participants supplemented with HSSD. HSSD supplementation significantly increased the levels of methyl palmitate, SM (d22:0/15:1), PC (18:5e/18:5), and 3-(3,4,5-trimethoxyphenyl) propanoic acid on day 14 after a third CVS vaccine dose. Previous studies have shown that methyl palmitate has anti-inflammatory and anti-fibrosis activities in organs such as lung and liver (45–47). Sphingolipid compounds have shown to counteract pulmonary fibrosis, alleviate chronic cardiac insufficiency, and influence atherosclerosis development (48). A clinical study has shown that the Xiaotan Jiangzhi formula of TCM combined with polyene phosphatidylcholine can help treat non-alcoholic fatty liver disease (49). 3-(3,4,5-trimethoxyphenyl)propanoic acid was reported to have anti-parasitic properties (50). Main pathways enriched in metabolites differentially regulated by HSSD were related to purine metabolism, vitamin B6 metabolism, folate biosynthesis, arginine and proline metabolism, and steroid hormone biosynthesis. Several vitamins have immunomodulatory functions on innate and adaptive immune responses to viral infection (51). Clinical studies have shown that vitamins have potential use as adjuvants for COVID-19 vaccines, and can assist in increasing the production of antibodies after immunization (52). Folic acid plays a crucial role in basic cellular processes, including nucleic acid biosynthesis, methyl group biogenesis, and amino acid metabolism, and has therefore potential in guiding the development of new drugs against infectious diseases (53). Immune cells are sensitive to arginine, which is closely related to cell growth, proliferation, and function, and plays an important regulatory role in the progression of chronic inflammation (54). It is thus proposed that HSSD boosts the immunogenicity of the CVS vaccine by enhancing the innate and adaptive immune responses through differential modulation of the synthesis and metabolism of vitamins, phospholipids, and amino acids involved in anti-inflammatory processes, providing enhanced organ protection and reducing the risk of SARS-CoV-2 infection.

Clinical reports demonstrated the efficacy and safety of the CVS vaccine in children, adults, and people over 65 years old with or without underlying diseases (55, 56). The AEs recorded after the third dose of the CVS vaccine in this trial were similar to those previously reported, and the absence of serious adverse reactions indicated that the vaccine was safe. Accordingly, the evaluation of safety indexes of AEs through analyses of liver function, renal function, FBG, and routine blood and urine screening confirmed that the CVS vaccine, the placebo, and HSSD were safe.

In conclusion, this trial showed that HSSD can enhance the immunogenicity of a third CVS vaccine dose in adults by stimulating humoral and cellular immunity, inhibiting the production of pro-inflammatory factors, and boosting SARS-CoV-2 antibody titers. To confirm the ability of HSSD to improve the immunizing effects of vaccines against COVID-19 and other diseases, clinical trials involving larger and more diverse populations are warranted.

## 5 Conclusions

We conducted a randomized, double-blind, placebo-controlled clinical trial to investigate the immunogenicity boosting effect of oral HSSD upon a third dose of CVS vaccine in adult participants. The results showed that HSSD can increase seroconversion, reflected by enhanced SARS-CoV-2-specific IgG antibody titers in serum. This boosting effect was likely mediated by increased production of IgG, IgM, and complement (C3 and C4) components, reduced synthesis of pro-inflammation factors (IL-6), and balancing of cellular immunity through normalized distribution and absolute counts of lymphocyte subsets. Serum untargeted metabolomics results suggested that the mechanisms by which HSSD enhanced the immunogenicity of the CVS vaccine were likely related to differential regulation of purine metabolism, vitamin B6 metabolism, folate biosynthesis, arginine and proline metabolism, and steroid hormone biosynthesis, among other metabolic pathways. In addition, the results of the questionnaire on AEs, as well as the evaluations of liver function, renal function, FBG, and routine blood and urine testing demonstrated the safety of oral supplementation with HSSD. This study provides reference and clinical basis for the use of HSSD to increase the immunogenicity of the CVS vaccine, and could set precedence for further studies assessing the pro-immunogenic potential of TCM preparations when administered in combination with COVID-19 vaccines.

## Data availability statement

The original contributions presented in the study are included in the article/**Supplementary Materials**, further inquiries can be directed to the corresponding author/s.

## Ethics statement

The study was approved by the Ethics Committee of Beijing University of Chinese Medicine (approval number 2021BZYLL0409). The studies were conducted in accordance with the local legislation and institutional requirements. The participants provided their written informed consent to participate in this study. All animal experiments were performed according to protocols approved by the Welfare and Ethical Inspection in the Beijing University of Chinese Medicine Animal Care Committee (No. BUCM-4-2021062901-2073). The study was conducted in accordance with the local legislation and institutional requirements.

## Author contributions

RYT: Investigation, Project administration, Supervision, Writing – original draft, Writing – review & editing. LW: Funding acquisition, Methodology, Writing – review & editing. JZ: Methodology, Writing – review & editing. WF: Formal analysis, Methodology, Validation, Writing – review & editing. RZ: Formal analysis, Methodology, Validation, Writing – original draft. JL: Project administration, Writing – review & editing. ML: Project administration, Writing – review & editing. MW: Project administration, Writing – review & editing. RL: Data curation, Project administration, Writing – review & editing. HN: Data curation, Project administration, Writing – review & editing. RT: Project administration, Writing – review & editing. YWC: Project administration, Writing – review & editing. YC: Project administration, Writing – review & editing. YJ: Project administration, Writing – review & editing. HZ: Project administration, Writing – review & editing.
