# Supplementary material for: Boosting the immunogenicity of the CoronaVac SARS-CoV-2 inactivated vaccine with Huoxiang Suling Shuanghua Decoction: a randomized, double-blind, placebo-controlled study
Source: Front Immunol. 2024 Apr 3;15:1298471. doi: 10.3389/fimmu.2024.1298471 (PMC11021573; doi:10.3389/fimmu.2024.1298471)
Supplement: Supplementary file 1 [file DataSheet_1.pdf]

Supplementary Material 1

HSSD boosting the vaccine-elicited immunogenicity of the S-RBD recombinant protein vaccine in mice

1. Materials and methods

1.1. Reagent and drug

Mouse Anti-Novel Coronavirus (2019-nCoV) S-RBD Protein IgG Antibody Detection Kit (Lot number: NCOmG20210902B, Beijing Wantai Bio-Pharmaceutical Co., LTD.), mCD4-R711-FITC (HQ12JL2501, Beijing Yiqiao Shenzhou Technology Co., LTD.), Sterile PBS solution (Hyclone, USA), Brilliant Violet 785™ Anti-Mouse CD8a (B344512, BioLegend), APC Anti-Mouse CD45 (B293308, BioLegend), Brilliant Violet 510 anti-mouse CD3e (B340821, BioLegend), Brilliant Stain Buffer Plus (0309780, BD), BD FACSTM Lysing Solution (9224600, BD), Anti-Rat and Anti-Hamster Ig/Negative Control (0022191, BD), BSA (210418, Salt City Cyberol). SARS-CoV-2 (2019-nCoV) Spike Recombinant recombinant Protein (S-RBD recombinant protein, lot number: 40592-V02H, Yiqiao Shenzhou Biotechnology Co., LTD.), Imject™ Alum Adjuvant (Lot number: C07-01013, Beijing Boosen Biotechnology Co., LTD.), HSSD (Lot number: 210101, Tianjin Zhongwei Hezhi Pharmaceutical Co., LTD.).

1.2. Animals and immunization protocol

Female BALB/c mice (8 weeks old, 18 - 20 g, certificate no. SCXK [Jing] 2011–0004) were obtained from SPF (Beijing) Biotechnology Co., Ltd, and fed in a standard animal room (room temperature: 20 °C - 24 °C, relative humidity: 30 % - 40 % and light condition: 12 h dark/light cycle). Mice were housed in the environment satisfied the National 137 Standards of Laboratory Animal Requirements (GB 14925-2001) of China. All animal experiments were performed according to protocols approved by the Welfare and Ethical Inspection in the Beijing University of Chinese Medicine Animal Care Committee (No. BUCM-4-2021062901-2073). A total of 36 mice were randomly divided into six groups (*n* = 6) and the used doses administration were as followings. Blank group (Control group), S-RBD recombinant protein immunization group (S group, 40 μg/single), S-RBD recombinant protein immunization + Aluminum adjuvant group (S + A group, 40 μg/single + aluminum adjuvant 200 μg/single), S-RBD recombinant protein immunization + HSSD low dose group (S + HSSD-L group, 40 μg/single + HSSD 0.465 g/kg/day, 5 times clinical dosage), S-RBD recombinant protein immunization + HSSD medium dose group (S + HSSD-M group, 40 μg/single + HSSD 0.930 g/kg/day, 10 times clinical dosage),

S-RBD recombinant protein immunization + HSSD high dose group (S + HSSD-H group, 10 times clinical dosage) 40 μg/single + HSSD 1.860 g/kg/day, 20 times clinical dosage). Mice were immunized by intramuscular injection at an intramuscular dose of 0.2 mL per mice.

A schematic diagram of vaccination and blood sample collection were shown in **Figure S1**. Blood collection of every group was performed on the first day, on the third week, on the seventh week, on the eleventh week, and on the thirteenth week, respectively. Besides, mice were vaccination for the first time on the first day, vaccination for the second time on the third week, and vaccination for the third time on the ninth week, respectively. Mice in the Control group were immunized by intramuscular injection with sterile PBS solution (0.2 mL/single), and mice in the other groups were immunized by intramuscular injection with S-RBD recombinant protein solution (containing S-RBD recombinant protein 40 μg/single) respectively. Among them, mice in S + A group were were immunized by intramuscular injection with S-RBD recombinant protein solution mixed of aluminum adjuvant solution. On the day after the every vaccination, mice in Control group and S + A group were intragastrically given distilled water for 7 days, while mice in S + HSSD-L, S + HSSD-M and S + HSSD-H groups were intragastrically given HSSD of corresponding concentration for 7 days. Corresponding biological samples were collected and stored in -80 ℃ refrigerator for testing. The obtained blood samples were kept for 2-3 h each time, centrifuged at 3500 r/min for 15 min, and the upper serum was absorbed and stored in the refrigerator at -80 ℃ after sorting.

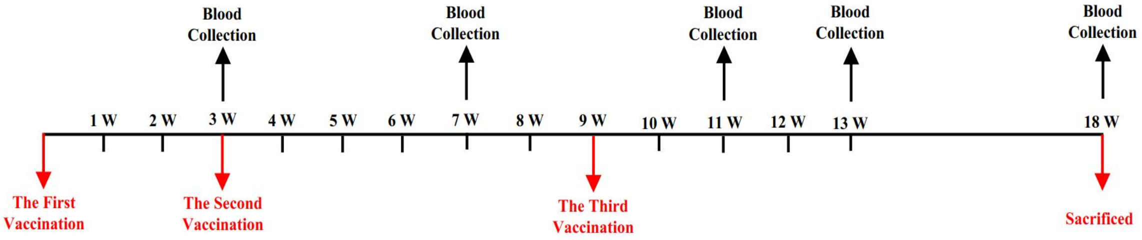

Figure S1: A schematic diagram of vaccination and blood sample collection of mice experiment. (Annotation: W means Week.)

1.3. Body weight recorded, serum anti-RBD IgG titer detection and lymphocyte immunophenotype by flow cytometry

Body weight, serum anti-RBD IgG titer in mice of every group were recorded and detected on the third week, on the seventh week, on the eleventh week, and on the thirteenth week, respectively. Finally, Plasma T cell subsets of $CD3^+$, $CD4^+$ and $CD8^+$ were measured by flow cytometry on the day of mice sacrificed after the third vaccination.

2. Statistical analysis

SPSS Statistics 20.0 was used for statistical analysis. The measurement data conforming to normal distribution were expressed as Mean ± Standard Deviation, comparison were performed by a one-way analysis of variance (ANOVA) followed by the Least-Significant Difference (LSD) post hoc test or Dunnett T3 test. Geometric Mean Titer (GMT) was calculated for antibody titers in each group. P value < 0.05 was considered statistically significant.

3. Results of HSSD boosting the vaccine-elicited immunogenicity of the S-RBD recombinant protein vaccine in mice

3.1. Body weight changes and serum anti-RBD IgG titer changes in mice

Results shown in **Figure S2 A**, at the 21st day after the first vaccination, the 28th day after the second vaccination, the 14th day after the third vaccination and the 28th day after the third vaccination, the body weight of mice in each group increased day by day, and there was no statistical difference in body weight among all groups (P > 0.05), these results indicating that S-RBD recombinant protein, aluminum adjuvant and HSSD had no adverse effect on inhibiting the body weight of mice. Results shown in **Figure S2 B - S2 E**, GMT (1:) of serum anti-RBD IgG titer in S group mice at day 21 after the first vaccination (Figure 3 A), day 28 after the second vaccination (Figure 3 B), day 14 after the third vaccination (Figure 3 C) and day 28 after the third vaccination (Figure 3 D) were 104, 3610, 19204 and 241017, respectively. S-RBD protein showed a gradual accumulation of immunogenicity in the three immunizations of mice. Besides, compared with the S group, the serum anti-RBD IgG titer in S + A group mice were significantly increased at day 14 and day 28 after the third immunization (P < 0.01), and the GMT (1:) in the four quartic time node were 819, 17711, 73826, 641356, indicating that aluminum adjuvant can significantly boosting the vaccine-elicited immunogenicity of the S-RBD recombinant protein vaccine. Otherwise, compared with the S group, the serum anti-RBD IgG titer in different HSSD dose groups assisted to boosting the antibody titer to varying degrees at day 21 after the first vaccination, day 28 after the second vaccination, day 14 and day 28 after the third vaccination, respectively (P < 0.05, P < 0.01, P < 0.001). The GMT (1:) of the four test points in S + HSSD-L group were 102, 3499, 25195 and 289913, respectively. The GMT (1:) of the four test points in S + HSSD-M group were 1540, 23961, 66214, 262436, respectively. The GMT (1:) of the four test points in S + HSSD-H group were 4263, 39783, 62798 and 473414, respectively. These results indicated that HSSD can significantly boosting the vaccine-elicited immunogenicity of the S-RBD recombinant protein vaccine.

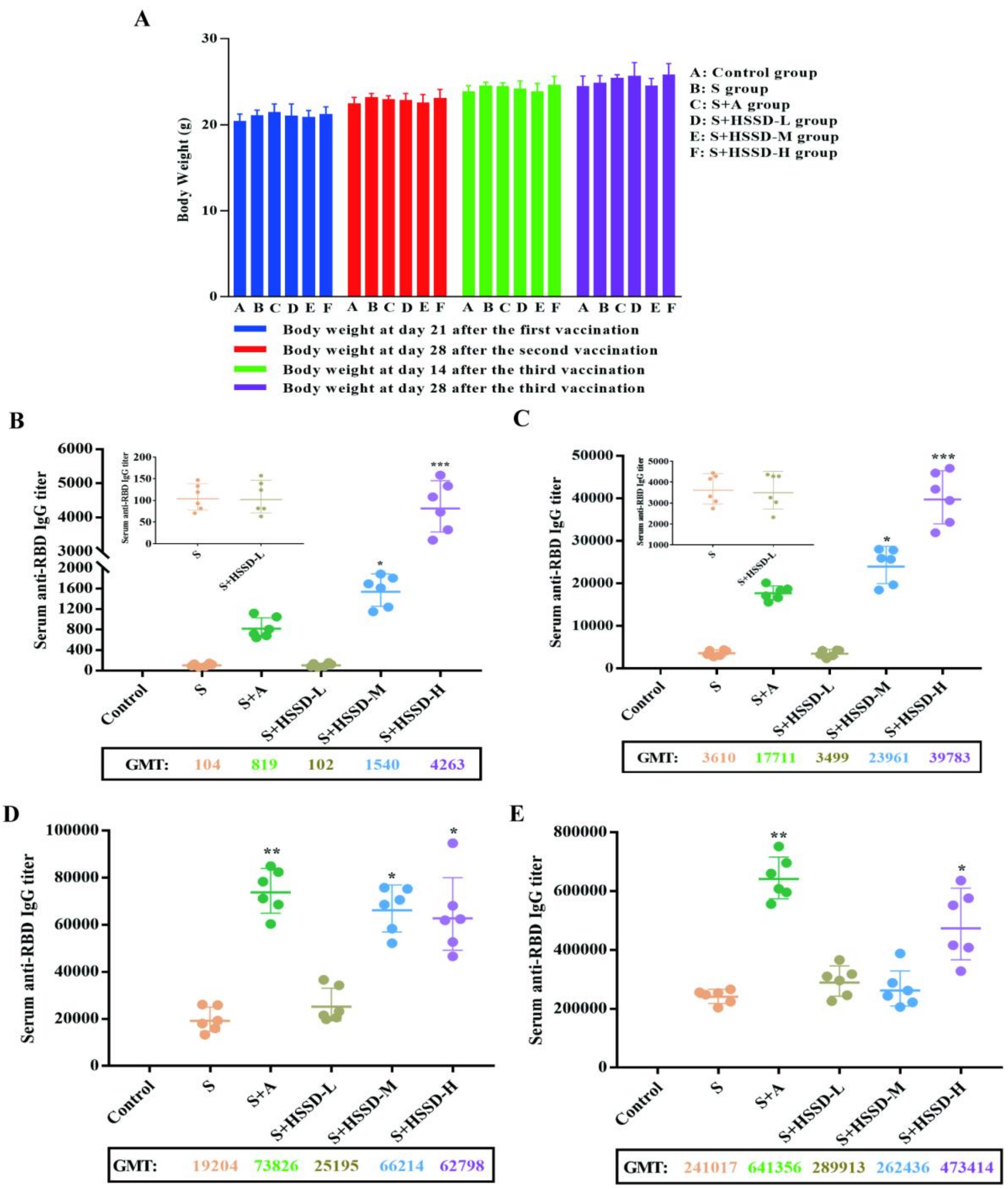

Figure S2: Body weight and serum anti-RBD IgG titer of mice after vaccination

(A) Body weight of mice, (B) Serum anti-RBD IgG titer and its GMT at day 21 after the first vaccination, (C) Serum anti-RBD IgG titer and its GMT at day 28 after the second vaccination, (D) Serum anti-RBD IgG titer and its GMT at day 14 after the third vaccination, and (E) Serum anti-RBD IgG titer and its GMT at day 28 after the third vaccination. Data were presented as Geometric Mean ± Geometric SD, n = 6, $^{*}P < 0.05$, $^{**}P < 0.01$, $^{***}P < 0.001$: significantly differently from the S group.

3.2. T lymphocyte immunophenotype changes in mice

Results shown in **Figure S3**, compared with the Control group, peripheral blood lymphocytes percentage of $CD3^+$, $CD4^+$ and $CD4^+/CD8^+$ ratio were significantly increased in S + A group and HSSD different dose groups mice ($P < 0.001$, $P < 0.01$, $P < 0.05$), while the percentage of $CD8^+$ was decreased (P < 0.05, P < 0.01). Besides, compared with S group, peripheral blood lymphocytes percentage of $CD3^+$, $CD4^+$ and

$CD4^+/CD8^+$ ratio were significantly increased in S + A group and HSSD different dose groups mice ($P < 0.001$, $P < 0.01$, $P < 0.05$), while the percentage of $CD8^+$ was decreased ($P < 0.05$, $P < 0.01$). Otherwise, representative flow cytometry plots of each detected T lymphocyte were shown in **Figure S4**.

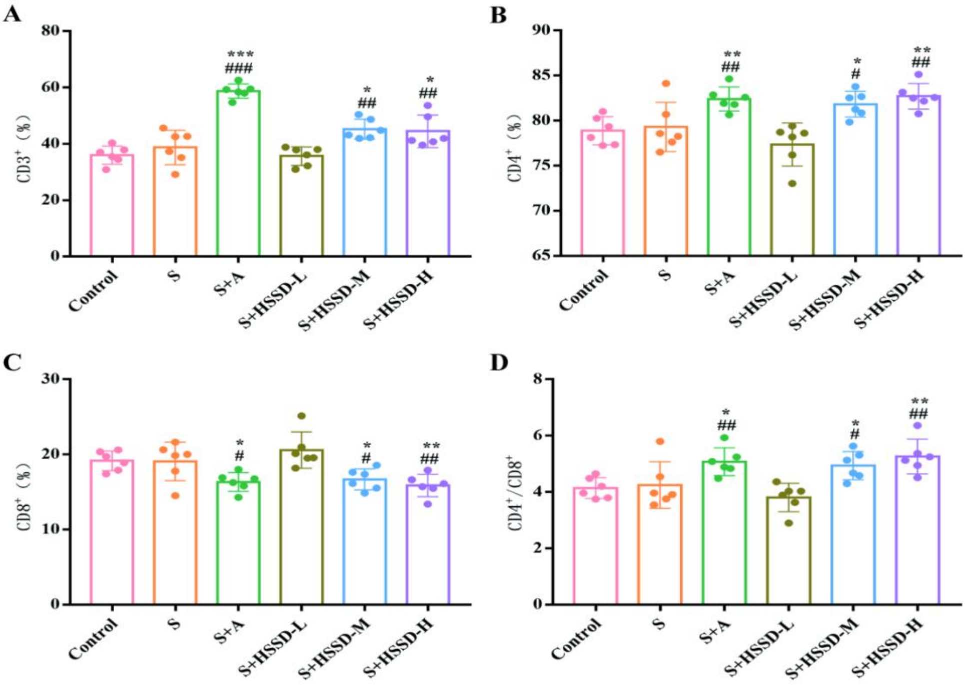

Figure S3: Percentage of $CD3^+$ T, $CD4^+$ T and $CD8^+$ T cells in peripheral blood of mice in after vaccination

(A) Percentage of $CD3^+$ T cells, (B) Percentage of $CD4^+$ T cells, (C) Percentage of $CD8^+$ T cells, (D) Ratio of $CD4^+/CD8^+$. Data were presented as Mean ± SD， $n = 6$, $^{\#}P < 0.05$， $^{\#\#}P < 0.01$， $^{\#\#\#}P < 0.001$: significantly differently from the Control group, $^{*}P < 0.05$， $^{**}P < 0.01$， $^{***}P < 0.001$: significantly differently from the S group.

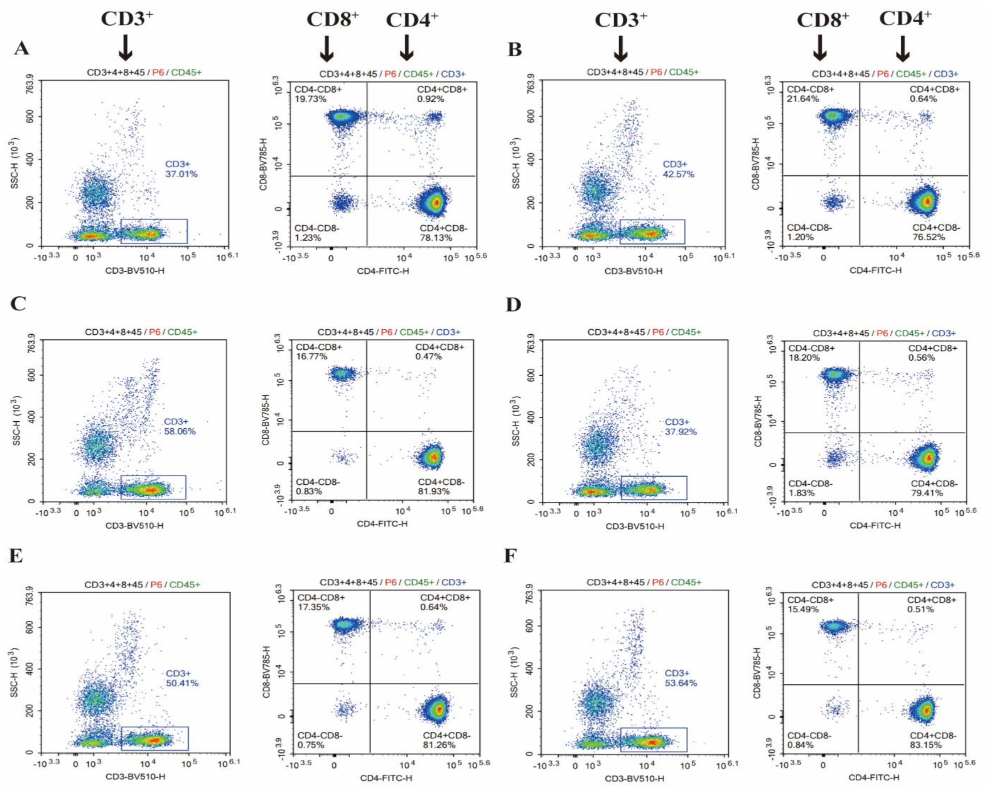

Figure S4: Representative flow cytometry plots of detected T lymphocyte

(A) Control group, (B) S group, (C) S + A group, (D) S + HSSD-L group, (E) S + HSSD-M group, (F) S + HSSD-H group.
