# Supplementary material for: Boosting the immunogenicity of the CoronaVac SARS-CoV-2 inactivated vaccine with Huoxiang Suling Shuanghua Decoction: a randomized, double-blind, placebo-controlled study
Source: Front Immunol. 2024 Apr 3;15:1298471. doi: 10.3389/fimmu.2024.1298471 (PMC11021573; doi:10.3389/fimmu.2024.1298471)
Supplement: Supplementary file 2 [file DataSheet_2.docx]

Supplementary Material 2

Table S1: Examination results of liver function, renal function, fasting blood-glucose, blood routine, and routine urine before and after third dose of CVS vaccine in all subjects.

| Term | CVS + Placebo group | CVS + HSSD group | *Z* value | *P* value |
| --- | --- | --- | --- | --- |
| Liver function test |  |  |  |  |
| 0 day: serum level of ALT (U/L) | 16.5 (14.5, 20) | 19 (14, 24) | - 0.916 | 0.36 |
| 14 day: serum level of ALT (U/L) | 15 (12.25, 19.5) | 15 (12, 23) | - 0.023 | 0.982 |
| *Z* value | - 1.291 | - 1.3 | - | - |
| *P* value | 0.197 | 0.194 | - | - |
| 0 day: serum level of AST (U/L) | 21.5 (20, 23.75) | 22 (19, 26) | - 0.509 | 0.611 |
| 14 day: serum level of AST (U/L) | 22 (20, 26) | 22 (19, 25) | - 0.312 | 0.755 |
| *Z* value | - 0.904 | - 0.018 | - | - |
| *P* value | 0.366 | 0.986 | - | - |
| Renal function test |  |  |  |  |
| 0 day: serum level of Crea (μmol/L) | 55.1 (51.7, 66.6) | 57.9 (51.7, 64.8) | - 0.752 | 0.452 |
| 14 day: serum level of Crea (μmol/L) | 55.2 (50.6, 68.4) | 57.1 (51.8, 66.1) | - 0.94 | 0.347 |
| *Z* value | - 0.082 | - 0.194 | - | - |
| *P* value | 0.934 | 0.846 | - | - |
| 0 day: serum level of BUN (mmol/L) | 4.3 (3.4, 5.2) | 4.1 (3.7, 5) | - 0.035 | 0.972 |
| 14 day: serum level of BUN (mmol/L) | 4.1 (3.6, 4.7) | 4.4 (3.8, 5.2) | - 0.917 | 0.359 |
| *Z* value | - 0.5 | - 0.441 | - | - |
| *P* value | 0.617 | 0.659 | - | - |
| Fasting blood-glucose (FBG) test |  |  |  |  |
| 0 day: serum level of FBG (mmol/L) | 4.8 (4.5, 5) | 4.8 (4.6, 5.1) | - 0.867 | 0.386 |
| 14 day: serum level of FBG (mmol/L) | 4.8 (4.5, 5) | 4.9 (4.7, 5.1) | - 1.659 | 0.097 |
| *Z* value | - 0.029 | - 0.892 | - | - |
| *P* value | 0.976 | 0.372 | - | - |
| Blood routine examination |  |  |  |  |
| 0 day: WBC (10^9^/L) | 6.18 (5.03, 7.03) | 5.82 (5.05, 6.76) | - 0.64 | 0.522 |
| 14 day: WBC (10^9^/L) | 5.8 (5.14, 6.99) | 5.55 (4.86, 6.87) | - 1.051 | 0.293 |
| *Z* value | - 0.64 | - 0.717 | - | - |
| *P* value | 0.522 | 0.474 | - | - |
| 0 day: RBC (10^9^/L) | 4.57 (4.29, 4.78) | 4.56 (4.31, 4.84) | - 0.194 | 0.846 |
| 14 day: RBC (10^9^/L) | 4.55 (4.23, 4.78) | 4.52 (4.34, 4.67) | - 0.059 | 0.953 |
| *Z* value | - 0.582 | - 0.376 | - | - |
| *P* value | 0.561 | 0.707 | - | - |
| 0 day: HGB (g/L) | 133 (129, 143) | 134 (129, 141) | - 0.223 | 0.823 |
| 14 day: HGB (g/L) | 133 (129, 142) | 135 (129, 142) | - 0.4 | 0.689 |
| *Z* value | - 0.3 | - 0.118 | - | - |
| *P* value | 0.764 | 0.906 | - | - |
| 0 day: PLT (10^9^/L) | 282 (220, 342) | 255 (229, 312) | - 1.116 | 0.264 |
| 14 day: PLT (10^9^/L) | 287 (241, 341) | 263 (228, 291) | - 1.821 | 0.069 |
| *Z* value | - 0.329 | - 0.082 | - | - |
| *P* value | 0.742 | 0.934 | - | - |
| 0 day: NEUT % (%) | 57.9 (52.1, 64.2) | 59.7 (51.5, 64) | - 0.112 | 0.911 |
| 14 day: NEUT % (%) | 57.7 (53, 64.5) | 56.1 (52.3, 63.1) | - 0.188 | 0.851 |
| *Z* value | - 0.206 | - 0.323 | - | - |
| *P* value | 0.837 | 0.747 | - | - |

Table S1: Examination results of liver function, renal function, fasting blood-glucose, blood routine, and routine urine before and after third dose of CVS vaccine in all subjects (Continuation of the above table 10).

| Term | CVS + Placebo group | CVS + HSSD group | *Z* value | *P* value |
| --- | --- | --- | --- | --- |
| Blood routine examination |  |  |  |  |
| 0 day: LYMP % (%) | 33.1 (27.8, 38.4) | 33.6 (28.6, 39.2) | - 0.07 | 0.944 |
| 14 day: LYMP % (%) | 34.6 (28.6, 38.2) | 35 (29.4, 39.9) | - 0.153 | 0.879 |
| *Z* value | - 0.07 | - 0.458 | - | - |
| *P* value | 0.944 | 0.647 | - | - |
| 0 day: MON % (%) | 5.6 (4.5, 6.8) | 5.5 (4.7, 6.5) | - 0.617 | 0.537 |
| 14 day: MON % (%) | 5.7 (5, 6.4) | 5.6 (5.1, 6.2) | - 0.553 | 0.581 |
| *Z* value | - 0.053 | - 0.1 | - | - |
| *P* value | 0.958 | 0.92 | - | - |
| Routine urine test |  |  |  |  |
| 0 day: SG of urine (g/mL) | 1.025 (1.02, 1.029) | 1.026 (1.0185, 1.0315) | - 0.531 | 0.595 |
| 14 day: SG of urine (g/mL) | 1.024 (1.015, 1.03) | 1.027 (1.0205, 1.031) | - 1.224 | 0.221 |
| *Z* value | - 0.197 | - 0.327 | - | - |
| *P* value | 0.844 | 0.743 | - | - |
| 0 day: pH of urine | 6 (5.5, 6.5) | 6 (5.5, 6.5) | - 0.312 | 0.755 |
| 14 day: pH of urine | 6 (5.5, 6.5) | 5.5 (5.5, 6) | - 0.56 | 0.575 |
| *Z* value | - 0.884 | - 1.206 | - | - |
| *P* value | 0.377 | 0.228 | - | - |
| 0 day: UF of urine | 20 (15.2, 24.7) | 22.6 (15.1, 26.85) | - 0.954 | 0.34 |
| 14 day: UF of urine | 21.5 (14.2, 25.1) | 20 (16.5, 26.3) | - 0.672 | 0.502 |
| *Z* value | 0 | - 0.167 | - | - |
| *P* value | 1 | 0.868 | - | - |
| 0 day: UBG negative cases | 35 (100 %) | 35 (100 %) | - | - |
| 14 day: UBG negative cases | 35 (100 %) | 35 (100 %) | - | - |
| 0 day: GLU negative cases | 35 (100 %) | 35 (100 %) | - | - |
| 14 day: GLU negative cases | 35 (100 %) | 35 (100 %) | - | - |
| 0 day: KET negative cases | 35 (100 %) | 35 (100 %) | - | - |
| 14 day: KET negative cases | 35 (100 %) | 35 (100 %) | - | - |
| 0 day: PRO negative cases | 35 (100 %) | 35 (100 %) | - | - |
| 14 day: PRO negative cases | 35 (100 %) | 35 (100 %) | - | - |

0 day: one day before the third dose of CVS vaccine, 14 day: on day 14 after the third dose of CVS vaccine. Data were presented as *M (P_25_, P_75_)* or *n* (%), *N* = 35, the normal range values of each index were as followings. Normal rang of ALT: 9 - 25 U/L, Normal rang of AST: 14 - 36 U/L, Normal rang of Crea: 46 - 92 μmol/L (female) and 58 - 110 μmol/L (male), Normal rang of BUN: 2.5 - 6.1 mmol/L (female) and 3.2 - 7.1 mmol/L (male), Normal rang of FBG: 3.9 - 6.1 mmol/L, Normal rang of WBC: 3.5 - 9.5 10^9^/L, Normal rang of RBC: 3.8 - 5.1 10^9^/L (female) and 4.3 - 5.8 10^9^/L (male), Normal rang of HGB: 115 - 150 g/L (female) and 130 - 175 g/L (male), Normal rang of PLT: 125 - 350 10^9^/L, Normal rang of NEUT %: 40 - 75 %, Normal rang of LYMP %: 20 - 50 %, Normal rang of MON %: 3 - 10 %, Normal rang of SG in urine: 1.003 - 1.030 g/mL, Normal rang of pH in urine: 5.4 - 8.4, Normal rang of UF in urine: 3.0 - 39 Ms/cm, Normal statement of UBG in urine: negative, Normal statement of GLU in urine: negative, Normal statement of KET in urine: negative, Normal statement of PRO in urine: negative.
